# Response: Commentary: Real-world post-deployment performance of a novel machine learning-based digital health technology for skin lesion assessment and suggestions for post-market surveillance

**DOI:** 10.3389/fmed.2024.1388422

**Published:** 2024-05-02

**Authors:** Lucy Thomas, Chris Hyde, Daniel Mullarkey, Jack Greenhagh, Dilraj Kalsi, Justin Ko

**Affiliations:** ^1^Chelsea and Westminster Hospital NHS Foundation Trust, London, United Kingdom; ^2^Exeter Test Group, Department of Health and Community Science, University of Exeter Medical School, Exeter, United Kingdom; ^3^Skin Analytics Ltd., London, United Kingdom; ^4^Department of Dermatology, School of Medicine, Stanford University, Stanford, CA, United States

**Keywords:** artificial intelligence, skin cancer, AI for skin cancer, AI as a medical device, DERM, deep ensemble for the recognition of malignancy, Skin Analytics

We thank Anderson et al. for their interest in and commentary on our paper. Real world evidence gathered through post-market surveillance (PMS) provides the opportunity to assess and monitor the performance of novel technologies in live clinical environments, outside of the limitations and constraints of traditional study designs. There is an emerging body of evidence to suggest that there can be a drop off in the performance of AI as a medical device (AIaMD) technologies when these progress from trial/study settings into real world deployments. Therefore, while service evaluation reporting for AIaMD deployments remains a relatively new exercise, we believe that transparent and candid publication of PMS data is an essential component of the evidence base underpinning these technologies. As a result, we welcome the opportunity to address the issues raised and clarify any misunderstandings. We hope that our response will help others feel more comfortable interpreting AIaMD service evaluation and PMS data going forwards.

## Response to “potential selection bias”

The commentary authors raise concerns around “the possibility of potential selection bias”. Their concerns relate to: (1) a lack of clarity about cases which were not able to be assessed by DERM, and (2) a mistaken belief that cases “where the final diagnosis is still pending” were considered benign within the case level analysis.

The original article details the eligibility criteria for assessment by DERM in Table 1. In the pathways described, trained healthcare professionals confirm which lesions are suitable for assessment by DERM; any lesions meeting DERM's pre-defined exclusion criteria are routed directly to teledermatology review. This is not selection bias: DERM is a UKCA Class IIa medical device with a specific scope of intended use. All licensed medical devices have exclusion criteria; these act as an important risk mitigation to ensure that the device is only used where it is appropriate to do so. In our publication, we have reported on the performance of DERM in line with its intended use based on all available data at the time of the analysis. All cases deemed ineligible for DERM assessment are accounted for within the “Not assessed by DERM” arm of Figure 3, along with any cases where technical issues prevented a DERM assessment from taking place. We have provided additional detail as to why these were not assessed by DERM in Table 1.

**Table 1:** Breakdown of reasons where lesions were not assessed by DERM.

**Reason not assessed by DERM**	**UHB-vA**	**WSFT-vA**	**UHB-vB**	**WSFT-vB**	**Total**
**Lesion not suitable for DERM assessment according to its intended use**					
Lesion too large for dermoscope	408	27	301	50	786
Open or ulcerated lesion	466	20	184	23	693
Hair, tattoos, scars	232	41	220	95	588
Network or image quality issues	296	27	157	18	498
Nails, mucosal or acral surfaces	232	22	150	31	435
Multiple exclusions	250	23	115	15	403
Unable to capture dermoscopic image	67	13	66	7	153
Previous biopsy site	6	1	4	0	11
Not a skin lesion	0	0	0	0	0
**Lesion not suitable for teledermatology pathway as a whole**				
Genital location	5	0	3	0	8
Patient aged under 18	0	0	0	0	0
Total	1,962	174	1,200	239	3,575

## Response to “attrition bias”

The second issue relates to cases “where the final diagnosis is still pending”. This is a challenge encountered in all post-market surveillance activities; any timely service evaluation will have incomplete data as not all cases will have a final outcome at the point of analysis. This reflects the real world diagnostic journey: patients may wait several days or weeks between their first assessment and subsequent appointments. For patients with lesions suspicious for malignancy, there are additional delays while waiting for a biopsy and subsequent histology reports. However, when conducting a service evaluation, there is the need to “lock the dataset” to run the analysis up to a specified end date. This poses an interesting challenge when it comes to publishing PMS outcomes in one-off academic manuscripts. To account for this, we present the impact at a case level, reporting on consecutive case series data for discharge and referral rates. However, we want to explicitly highlight that the commentary is wrong to conclude that any patient awaiting a procedure or histology outcome “would have erroneously appeared as benign”. This is incorrect: these cases are specifically accounted for as “Non-discharge” lesions in Figure 3. In the per lesion analysis, the analysis is only conducted on lesions with a ground truth diagnosis of a biopsy-confirmed cancer or a biopsy/clinically-confirmed pre-malignant or benign lesion; this is stated within the opening paragraph of the results section. We do agree that with respect to the lesion based accuracy estimates there is potential for attrition bias, particularly in the second data collection period and that it is possible that with more complete follow-up further missed cases of cancer may emerge. We can however be reassured that the sensitivity of DERM in the first data collection period, not affected by attrition bias, is also strong.

We welcome constructive recommendations on how others might approach the issue of pending results, especially when there is a need for service evaluation reporting to remain proximate to the time of analysis. We discuss our thoughts on improving the validity of PMS in the future in the section entitled “Considerations arising from assessment of openness to bias”. We suggest, “Careful attention to documenting and describing legitimate losses to follow-up, patients who are ineligible for assessment and technical failures…” which is designed to address the risk of attrition bias. Furthermore, as outlined in our paper, we continue to prospectively monitor DERM's performance as part of our rigorous PMS strategy. By generating quarterly reports that are shared with our partners, we continually update the data set to ensure that all cases receive a final ground truth outcome at the earliest possible opportunity.

## Response on cancer prevalence rates

The commentary raises questions around the prevalence of skin cancer seen in the populations at both sites and expresses surprise at the difference encountered with each version of DERM. Anderson et al. reference a questionnaire-based study from 2004 which suggests that up to 12% of urgent suspected skin cancer referrals result in a melanoma, SCC or rare skin cancer diagnosis. In reality, the true conversion rate sits between 6.5 and 8.5%, as per UK National Disease Registration Service data from 2009 to 2021^1^. In our paper, the overall conversion rate was 5.7%. Looking specifically at the local populations in Birmingham and West Suffolk between 2009 and 2021, skin cancer conversion rates fluctuate between 4.2–9.6% and 7.0–9.9%^1^ respectively, as compared with 4.2% and 6.2% in our study. BCC data is less well reported and only recently has some data been made available from national NHS sources (1). This makes it difficult to baseline how many BCCs are expected in urgent skin cancer referral populations.

The slightly lower prevalence of melanoma, SCC and rare skin cancers seen in our study reflects the inevitable delays in confirming ground truth outcomes for patients sent for biopsy. Indeed, the apparent “drop” in skin cancer prevalence noted between earlier DERM-vA and later DERM-vB deployments adds weight to this conclusion as, at any point in time, there will be cases with pending ground truth outcomes. As noted above, sources of real world delay include surgical capacity, pathologist workforce availability and histology data gathering. Inevitably then, older cases are more likely to have ground truth outcomes and this is reflected in the chronology of our data set. As previously indicated, however, we do note that a small minority of cases clinically diagnosed as pre-malignant or benign on will have their final diagnosis changed following biopsy. This highlights the need for post-market surveillance to be an ongoing process in order to present the most up-to-date picture possible. Needless to say, all known cancers at the time of the study were reported in our paper.

Another possible source of perhaps lower cancer prevalence seen is that any clinically diagnosed premalignant or benign lesion is at risk of validation bias. Given that the sensitivity for malignancy of teledermatology workflows is estimated at 94.9% (2), we might expect 1 in 20 cancers to be assigned a false negative clinical diagnosis. Therefore, the true prevalence of skin cancer would likely be higher if all lesions referred on an urgent suspected cancer pathway were biopsied.

## Response on atypical and dysplastic naevi

We appreciate the highlighting of atypical naevi by the commentary authors. These are a clinically gray area and, in co-designing pathways with both UHB and WSFT partner sites, lesions assessed as atypical naevi by DERM remained on the urgent suspected cancer referral pathway. Underpinning the challenge of atypical naevi is a degree of diagnostic uncertainty, even with histology-based outcomes. For example, a large BMJ study suggested that histology diagnoses spanning moderately dysplastic naevi to early stage invasive melanoma are neither reproducible nor accurate (3). However, we stand by our overall specificity estimates for benign lesions (70.1–73.4% with DERM-vB): given that all cases were taken from a post-referral population (i.e. when patients have been referred by primary care practitioners on an urgent suspected cancer pathway), DERM's ability to diagnose benign lesions with a high degree of accuracy allows these patients to be safely discharged, thus relieving pressures on secondary care.

## Conclusions

Our paper demonstrates that DERM's real world performance exceeded pre-specified sensitivity targets of at least 95% for melanoma and squamous cell carcinoma (SCC) and 90% for basal cell carcinoma (BCC), intraepidermal carcinoma (IEC) and actinic keratosis. The additional observations on possible bias, although partially correct with respect to attrition bias, do not substantially undermine the results. We highlight our data is based upon 14,500 patients and 8,500 lesions with ground truth outcomes, including over 1,100 confirmed malignancies. To put this volume of evidence into context, this is 3,000 more lesions than were included in the Cochrane review of teledermatology (2) and teledermatology has been endorsed nationally in the UK (4). Furthermore, we continue to monitor the performance of DERM as part of our PMS strategy and our most recent quarterly report (based upon ~30,000 lesions with a final diagnosis across 11 NHS deployments) demonstrates that DERM continues to perform above these sensitivity targets.

We are disappointed by the authors' suggestion that we have displayed a lack of transparency and adequate scientific rigor. We are proud to share not only one of the first real world post-deployment service evaluations of an AIaMD but also our recommendations for how these technologies can be continually assessed and monitored in live clinical environments. We see our post-market surveillance as comprehensive and robust, surpassing existing standards of care in monitoring patient outcomes. We also continue to work with independent experts to validate our results, with one independent report validating our approach to post-market surveillance (5) and reports from two further independent teams expected shortly.

We hope that our responses help to address the concerns of the commentary authors and others interested in AIaMD technologies, demonstrating particularly that selection bias is not present and showing that the effect of attrition bias is not as dramatic as claimed. We welcome the opportunity for further dialogue to share our work and how we can continue to refine the PMS of AIaMDs.

## Author contributions

LT: Writing – review & editing. CH: Writing – review & editing. DM: Writing – original draft, Writing – review & editing. JG: Writing – review & editing. DK: Writing – original draft, Writing – review & editing. JK: Writing – review & editing.
